# Revisiting the flight dynamics of take-off of a butterfly: experiments and CFD simulations for a cabbage white butterfly

**DOI:** 10.1242/bio.059136

**Published:** 2022-03-24

**Authors:** Kosuke Suzuki, Masashi Nakamura, Masaya Kouji, Masato Yoshino

**Affiliations:** 1 Institute of Engineering, Academic Assembly, Shinshu University, Nagano 380-8553, Japan; 2 Department of Mechanical Systems Engineering, Faculty of Engineering, Shinshu University, Nagano 380-8553, Japan

**Keywords:** Flapping flight, Butterfly, Take-off, Computational fluid dynamics

## Abstract

We conducted measurements of the taking-off motion of a butterfly (*Pieris rapae*) and numerical simulations using a computational model reflecting its motion. The computational butterfly model is composed of a thorax, an abdomen, and four wings (left and right wings with fore and hind parts), i.e. a six-link, rigid-body system. The present model is more sophisticated than any models that have ever been constructed in existing studies. In the butterfly model, the body trajectory and thoracic pitching angle can be calculated from the equations of motion, whereas the abdominal angle and wings’ joint angles are prescribed by the measured data. We calculated the flow field, aerodynamic force and torque generated by the butterfly model using the immersed boundary–lattice Boltzmann method. As a result, the butterfly generates the horizontal vortex ring and aerodynamic lift force during the downstroke, while it generates the vertical vortex ring and aerodynamic thrust force during the upstroke. The leg impulsion is essential in the upward motion of the taking-off butterfly rather than the aerodynamic lift force by the flapping wings. The inertial forces of the abdomen and wings are comparable in magnitude with the aerodynamic forces, but the net influence of the inertial forces on the position of the butterfly is not significant due to the offsetting of the body and wing inertia. The net aerodynamic and gravitational torques raise the thorax of the butterfly, and the net inertial torques suppress the rise of the thorax.

## Abstract (precis)

**Summary:** We conducted measurements of the taking-off motion of a butterfly (*Pieris rapae*) and numerical simulations using a more sophisticated computational model than existing butterfly models.

## INTRODUCTION

Butterflies have been attracting people for a long time not only because of their beautiful wings but also because of their erratic flight, which is often described as ‘fluttery’. From a biological viewpoint, it has been suggested that their erratic flight was developed as an evolutionary response to aerial predation, i.e. for evading other insects or birds aiming at them (Dudley, 2002). From a physical viewpoint, their erratic flight should be related to their unique morphological and kinematic features, e.g. large variation in the inclination of the stroke plane (Sunada et al., 1993); low flapping frequency and broad wings (Shyy et al., 2013); low wing loading (Zheng et al., 2013); heavy weight of the wings relative to the total weight (Suzuki et al., 2019); abdominal oscillation (Chang et al., 2020); and the clapping motion of wings (Johansson and Henningsson, 2021). Thus, the erratic flight of butterflies is a result of the dynamical interactions between the body, wings, and ambient air.

To investigate the flight dynamics of butterflies, we must consider the coupled problem of the dynamics of the wing–body system as well as the aerodynamics. Many researchers have constructed and developed dynamical models of butterflies. Sunada et al. (1993) are the pioneers who constructed the dynamical model of a butterfly. Their model is a four-link, rigid-body system consisting of a thorax, an abdomen, and left and right wings, and it reflects the motions of the body and the wings of a butterfly (*Pieris melete*) in take-off flight. The aerodynamic force and torque acting on the model were estimated by the vortex method. As a result, it was found that vertical and horizontal aerodynamic forces are generated during the downstroke and the upstroke, respectively, due to the variation of the inclination of the stroke plane, which is the key mechanism of butterfly flight. In addition, it was found that the aerodynamic torque always raises the thorax, and the rise of the thorax is suppressed by the inertial torque of the abdomen.

Senda et al. (2012) also constructed a four-link, rigid-body system modeling a chestnut tiger butterfly (*Parantica sita*) in forward flight, and the aerodynamic force and torque were estimated by the panel method. Subsequently, the computational accuracies of the flow field and aerodynamic force and torque were improved by using the immersed boundary method (Yokoyama et al., 2013). As a result, it was found that the model can produce enough forces to achieve the flapping flight, but its flight was longitudinally unstable due to the increase in the pitching angle.

Bimbard et al. (2013) considered a butterfly model composed of two massless wings and a body represented by a mass point to investigate the take-off maneuver of a cabbage white butterfly (*Pieris rapae*). The flow field and aerodynamic force were calculated by using the Fourier pseudo-spectral method with the volume penalization method. In addition, they modeled the leg forces generated by the active extension of the legs. They found that not only the aerodynamic forces generated by the wings but also the leg forces are needed to faithfully reproduce the trajectory of the butterfly's body measured by the experiments.

Suzuki et al. (2015) constructed a butterfly-like flapping wing–body model composed of two massless wings and a body represented by thin square plates and a thin rod, respectively. This model is the simplest model which can move translationally and rotationally while flapping its wings downward and backward to generate lift and thrust forces, respectively. The flow field and aerodynamic force and torque were calculated by using the lattice Boltzmann method with the immersed boundary method. As a result, it was found that even this simple model can generate enough lift force to support an actual butterfly's weight. In addition, the pitching angle can be controlled by flexing at the joint between the thorax and abdomen. Subsequently, their model was extended such that the mass of the wings could be considered (Suzuki et al., 2019). As a result, it was found that the aerodynamic forces decrease as the wing-mass ratio (the ratio of the wing mass to the total mass) increases, since for a large wing-mass ratio the body has large vertical and horizontal oscillations in each stroke and consequently the speeds of the wing tip and leading edge relatively decrease.

Fei and Yang (2015, 2016) constructed a butterfly model composed of two massless wings and a body to investigate the forward flight of leaf butterflies (*Kallima inachus*). In this model, the relative motion of the abdomen is neglected, and the pitching motion of the body is prescribed. The flow field and aerodynamic force and torque were calculated by using the ANSYS FLUENT. They showed that the variation of the flapping speed is substantial in one flapping cycle and makes a large difference on the time-averaged aerodynamic forces compared with the flight with a constant speed, and that the flight mode is closely related to the pitching angle of the body. Recently, Chang et al. (2020) have extended their model to a four-link, rigid-body system modeling a paper-kite butterfly (*Idea leuconoe*) in forward flight, so that the wing mass and abdominal motion can be considered. In this model, the phase of the abdominal oscillation is tunable, whereas the pitching motion of the thorax is prescribed. As a result, it was found that the abdominal oscillation enhances the lift and thrust forces due to the translational motion of the joint between the thorax and the abdomen relative to the center of mass. In addition, it was found that the phase of the abdominal oscillation recorded from actual butterfly's flights maximizes the lift and thrust forces.

Tejaswi et al. (2021) constructed a four-link, rigid-body system modeling a monarch butterfly (*Danaus plexippus*). The aerodynamic force and torque were formulated as a function of the wing kinematics and body motion by using a quasi-steady blade element method. Thus, the flight dynamics of the monarch butterfly are formulated directly on the configuration manifold, while the pitching angle of the body is not included in the unknown coordinates but given as the harmonic oscillation with unknown parameters. By using this model, they reproduced the thorax and abdomen motions as well as the resultant forces consistent with the measured data. In addition, they showed that the abdomen oscillation not only results in a reduction of the energy and power consumption but also improves the stability of periodic orbits.

From the above review about dynamical models of butterflies, we find the importance of the dynamical interactions between the thorax, abdomen, wings, and ambient air in the flapping flight of butterflies. Especially, the effects of the wing mass (Suzuki et al., 2019) and abdominal oscillation (Chang et al., 2020) on the aerodynamic force and torque are essential to be considered in the investigation into the flight dynamics of butterflies. From this viewpoint, the conventional dynamical models of butterflies for take-off flight (Sunada et al., 1993; Bimbard et al., 2013) might be insufficient. The model of Sunada et al. (1993) is composed of a thorax, an abdomen, and left and right wings, but the equations of motion do not include a term related to the time derivative of the inertia moment of the wings, which should have a significant effect on the flight maneuvers of butterflies (Lin et al., 2012). The model of Bimbard et al. (2013) does not include either of the wing mass or the abdominal oscillation. Thus, it is worthwhile to revisit to the flight dynamics in the take-off of a butterfly by using a more sophisticated model including the wing mass and abdominal oscillation like the model of Senda et al. (2012); Yokoyama et al. (2013).

In the present study, we investigate the flight dynamics in the take-off of a cabbage white butterfly (*Pieris rapae*) by using a dynamical model composed of a thorax, an abdomen, and four wings (left and right wings with fore and hind parts), i.e. a six-link, rigid-body system. The joint angles between the thorax and abdomen and between the thorax and wings are given from the experimental measurement using high speed video cameras and a motion capturing system. The air flow around the butterfly model is calculated by using the immersed boundary–lattice Boltzmann method (Suzuki and Inamuro, 2011). We consider the motion of the butterfly model in the following four cases: (i) to prescribe both the translational and rotational motions by the experimental data; (ii) to calculate the translational motion of the model by assuming that all the masses of the model are concentrated at a reference point; (iii) to calculate the translational motion of the thorax from the equations of motion, whereas the rotational motion is prescribed; (iv) to calculate both the translational and rotational motions of the thorax from the full equations of motion. In case ii, where the equations of motion are the same as those in Bimbard et al. (2013) and we validate our simulations by checking whether the same conclusion as the study of Bimbard et al. (2013), can be reproduced. In case iii, we check the effects of the abdominal oscillation and wing mass on the translational motion of the body by comparing case ii and two subcases without and with wing mass (iii-a and iii-b, respectively). In case iv, we check the effects of the abdominal oscillation and wing mass on the rotational motion of the body by comparing case iii and two subcases without and with wing mass (iv-a and iv-b, respectively).

## RESULTS

### Case i: vortex structure and aerodynamic force

**Fig. 1.**
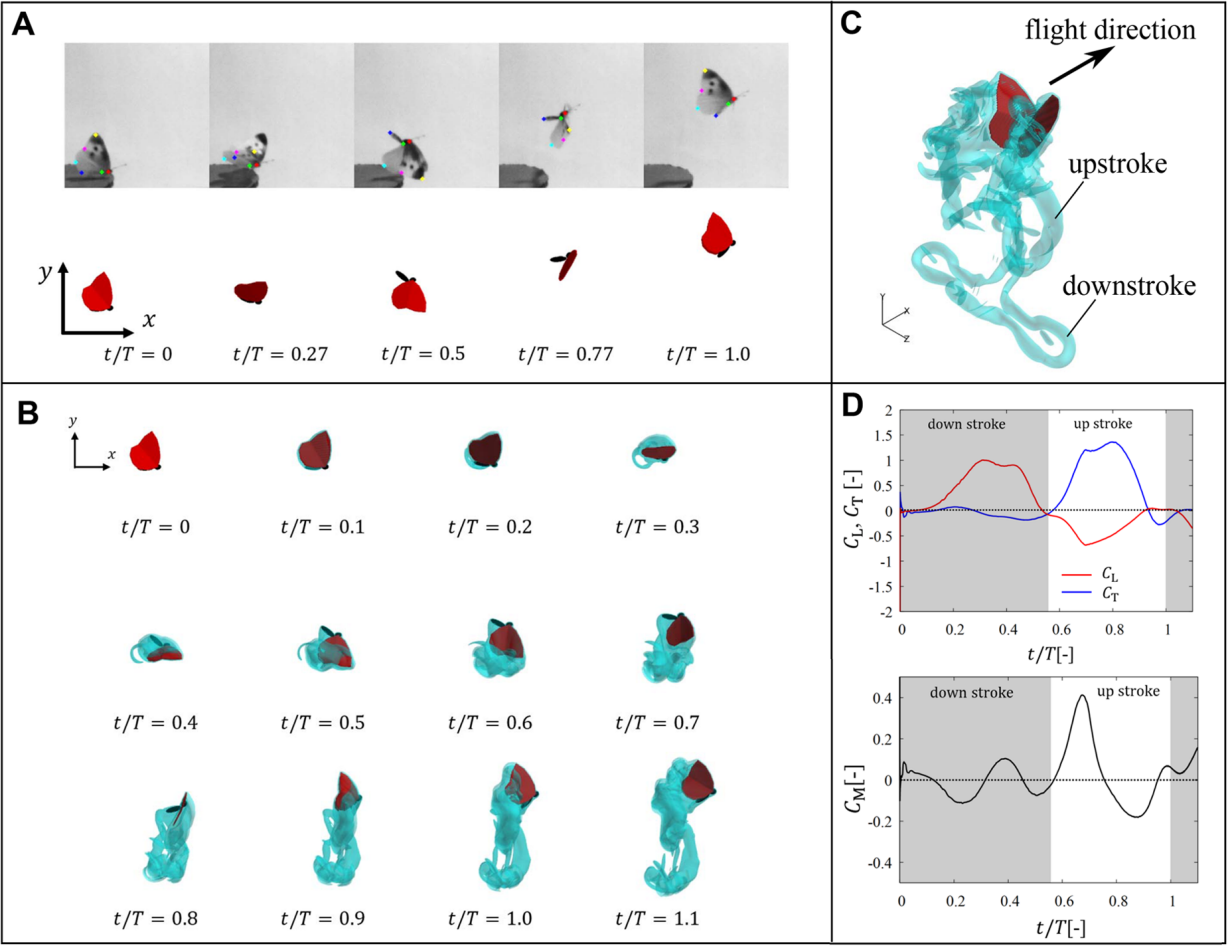
**Results in case i.** (A) Butterfly's motion in the take-off from *t*/*T*=0.0 to *t*/*T*=1.0 in the experiment and the simulation; (B) isosurface of the magnitude of the vorticity 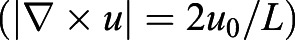 around the butterfly model from *t*/*T*=0 to *t*/*T*=1.1 viewed from the right side of the model; (C) vortex structure 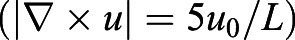 behind the butterfly model at *t*/*T*=1.1; (D) time variations of the lift coefficient *C*_L_, the thrust coefficient *C*_T_, and the pitching moment coefficient *C*_M_**.**

At first, we show the results in case i to observe the vortex structure and aerodynamic force generated by the butterfly in take-off. We can see from Fig. 1A and its corresponding movie (Movie 1) that the present model appropriately reproduces the motion of the actual butterfly. In addition, from Movie 1, we can see that the model wing is slightly flexed on the line which distinguishes the forewing and hindwing. However, the angle between the fore- and hindwings 
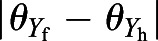
 is very small at most a few degrees, and our preliminary calculations when the fore- and hindwings are treated as a single whole wing (i.e. 
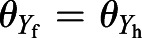
) give almost the same vortex structure and aerodynamic force as those in the present simulations.

Fig. 1B and its corresponding movie (Movie 2) show the vortex structure around the butterfly. We can see that the butterfly generates the vortex on the dorsal side of the wings during the downstroke and releases it downward in the beginning of the next upstroke. Also, the butterfly generates the vortex on the ventral side of the wings during the upstroke and releases it backward in the beginning of the next downstroke. Since the butterfly moves upward and forward as shown in Fig. 1B, it releases the generated vortices in the direction opposite to its traveling direction. As a result, the butterfly generates the horizontal and vertical vortex rings behind it during the downstroke and upstroke, respectively, as shown in Fig. 1C. A similar vortex structure was observed in the experimental visualization using a tomographic particle image velocimetry conducted by Johansson and Henningsson (2021).

Fig. 1D shows the aerodynamic lift coefficient 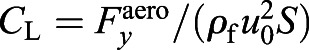, thrust coefficient 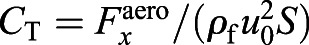, and pitching moment coefficient 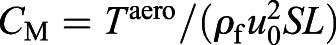. We can see from this figure that the butterfly generates lift force in the downstroke and thrust force in the upstroke. These aerodynamic forces are given by the unique wing motion that the butterfly flaps its wings downward in the downstroke and upward in the upstroke by changing the pitching angle, i.e. the inclination of the stroke plane (Sunada et al., 1993). Meanwhile, the pitching moment coefficient shows a complicated curve which alternates negative and positive peaks independently of downstroke and upstroke. This contradicts the result of Sunada et al. (1993) that the aerodynamic pitching moment is always positive and raises the thorax. This contradiction might be attributed to individual differences such as wing shape and wing kinematics. Thus, the aerodynamic pitching moment does not always raise the thorax in the take-off of a butterfly.

### Case ii: leg impulsion

Secondly, we show the results in case ii to check whether the same conclusion as the study of Bimbard et al. (2013) on the leg impulsion can be reproduced by the present simulations. In order to evaluate the effect of the leg impulsion, we consider the subcase without jump by the legs as well. In this subcase (referred to as ii without jump), we assume that the legs just support the weight of the butterfly until *t*/*T*=0.3. Thus, in the early stage of the simulation 0≤*t*/*T*≤0.3, we calculate the position *x* by cancelling the gravitational force, i.e. by adding the leg force + *MGe*_*y*_ to the right hand side of Eqn (14).

**Fig. 2.**
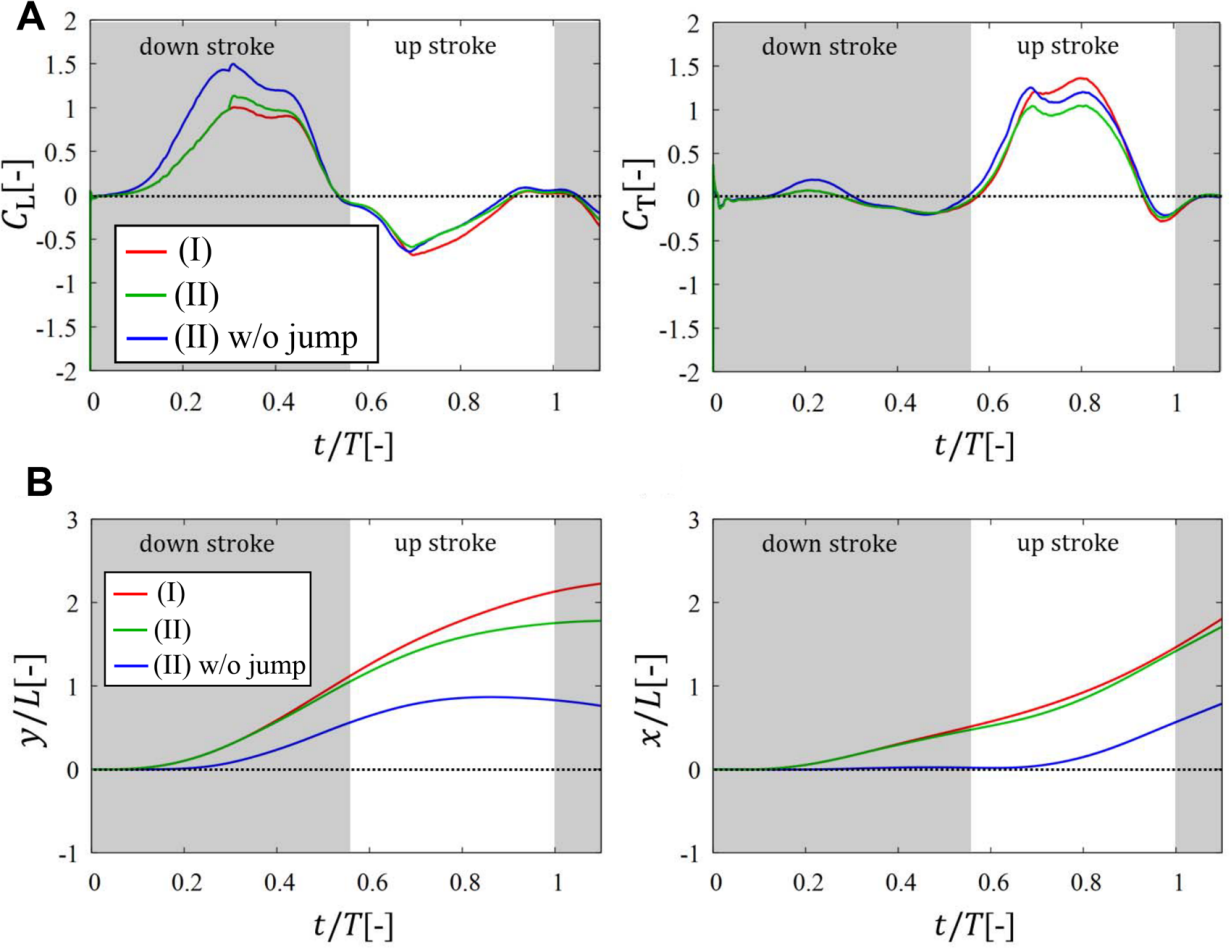
**Results in case ii.** Time variations of (A) the lift coefficient *C*_L_ and the thrust coefficient *C*_T_ and (B) the position of the reference point in the *y*-direction and the *x*-direction in cases i, ii, and ii without jump.

Fig. 2 shows the lift coefficient *C*_L_, thrust coefficient *C*_T_, vertical position *y*, and horizontal position *x*. We can see from the left panel of Fig. 2A that the lift forces in cases i and ii are comparable to each other, whereas the lift force in case ii without jump is significantly larger than the other cases in the downstroke. From the left panel of Fig. 2B, however, we can see that the vertical position in case ii without jump is significantly lower than the other cases throughout the one period. This means that the leg impulsion is crucial in the upward motion of the butterfly during take-off, and is greater in importance than lift from the flapping wings. Thus, the same conclusion as the study of Bimbard et al. (2013) is reproduced by the present simulations.

Also, we can see from the right panel of Fig. 2A that the thrust forces in cases i and ii without jump are comparable to each other, whereas the thrust force in case ii is smaller than the other cases in the upstroke. This difference in the thrust force between cases with/without jump is attributed to the fact that in case ii the wing speed in the backward direction decreases due to the forward speed given by the leg impulsion. From the right panel of Fig. 2B, however, we can see that the horizontal position in case ii without jump is significantly behind the other cases. Whereas in case ii without jump the horizontal position is almost zero since the aerodynamic thrust force is zero, in case ii the horizontal position increases not by the aerodynamic thrust force but by the initial momentum due to the leg impulsion. As shown in the above result, this butterfly takes off in the forward direction, while the butterfly in the study of Bimbard et al. (2013) takes off in the backward direction. This might be attributed to the fact that the individual in the present study takes off from the edge of the stand. In any case, we can conclude that the effect of the leg impulsion is significant even in the forward motion of the taking-off butterfly.

To make quantitative comparisons, we define the root mean square difference in a variable Ψ between two cases P and Q as follows:

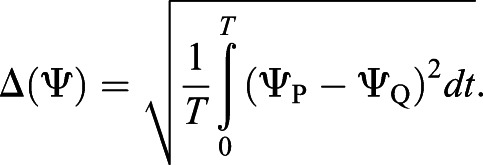


Table 1 includes the root mean square differences between cases i and ii and between case ii without jump and case i. From this table, we can confirm the above conclusions.

**Table 1.**
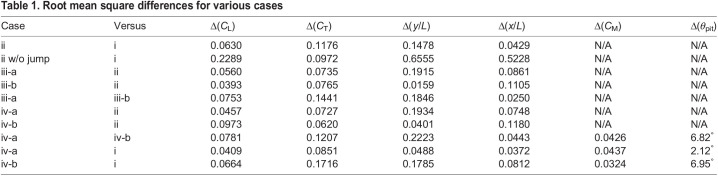
Root mean square differences for various cases

### Case iii: abdominal oscillation and wing mass

**Fig. 3.**
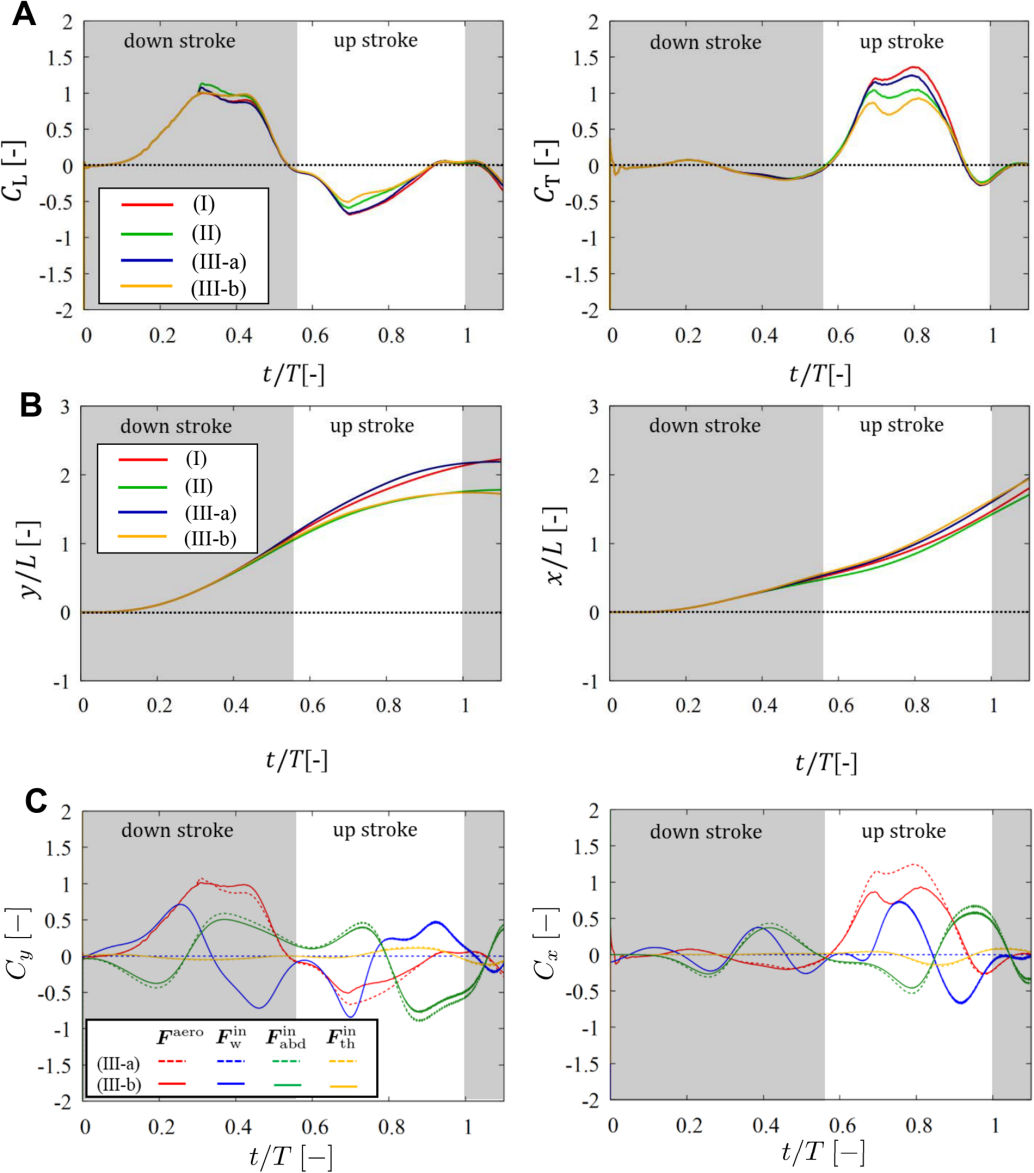
**Results in case iii.** Time variations of (A) the lift coefficient *C*_L_ and the thrust coefficient *C*_T_ and (B) the position of the reference point in the *y*-direction and the *x*-direction in cases i, ii, iii-a, and iii-b. (C) Comparison of the vertical- and horizontal-force coefficients *C*_*y*_ and *C*_*x*_ for the aerodynamic force ***F***^aero^ with those for the inertial force of the wings 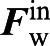, abdomen 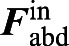, and thorax 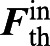 in cases iii-a and iii-b.

Thirdly, we show the results in case iii to check the effects of the abdominal oscillation and wing mass on the translational motion of the body. Case iii has two subcases iii-a and iii-b, which correspond to the cases without and with wing mass, respectively (see also Table 3). Fig. 3 shows the results of *C*_L_, *C*_T_, and ***x***=(*x*, *y*) in cases i, ii, iii-a, and iii-b. Also, this figure includes the comparison between the aerodynamic and inertial forces in cases iii-a and iii-b. The definition of the inertial forces is shown in the Appendix. We can see from the left panel of Fig. 3A that all cases are comparable to each other in terms of *C*_L_. From the left panel of Fig. 3B, however, we can see that the vertical position in case iii-a is slightly higher, by about *L*/2=10 mm at the end of upstroke, than that in case ii, and the vertical position in case iii-b is comparable to that in case ii. The quantitative differences between these cases can be seen in Table 1. The difference between cases ii and iii-a is derived from the existence of the inertial force of the body especially due to the abdominal oscillation, and the difference between cases iii-a and iii-b is derived from the existence of the inertial force of the wings. Thus, the upward motion of the butterfly is enhanced by the body inertia and deteriorated by the wing inertia, which is consistent with the conclusions by Chang et al. (2020) and Suzuki et al. (2019). Actually, from the left panel of Fig. 3C, the vertical inertial forces of the abdomen and wings are respectively positive and negative for 0.3<*t*/*T*<0.8. In addition, the effect of the body inertia on the upward motion is almost cancelled by the effect of the wing inertia, and consequently the vertical displacements in cases ii and iii-b almost coincide with each other. This is consistent with the results by Chang et al. (2020) that the inertial force of the wings has nearly the same magnitude but the opposite trend compared with that of the abdomen.

On the other hand, we can see from the right panel of Fig. 3A that the peak value of *C*_T_ is larger in order of i, iii-a, ii, and iii-b. This difference in *C*_T_ might be attributed to the difference in the vertical speed of the body. In the right panel of Fig. 3B, however, the difference in the horizontal displacements between these cases is not significant. In particular, the horizontal displacements in cases iii-a and iii-b almost coincide with each other (see also Table 1). This is because the inertial force of the wings compensates the decrease in the aerodynamic force as shown in the right panel of Fig. 3C.

As discussed above, the inertial forces of the abdomen and wings can have a significant effect on the translational motion of the butterfly. (The inertial force of the thorax is small enough to be ignored compared with the other inertial forces). Interestingly, however, the resultant trajectory in case ii without the inertial forces is comparable with that in case iii-b with the inertial forces. This is because the influence of the body inertia on both the upward and forward motions is almost cancelled by the influence of the wing inertia as shown in Fig. 3C. This result suggests that the translational motion of a butterfly within short duration after the take-off can be modelled without the inertial forces as in case ii.

### Case iv: pitching rotation

**Fig. 4.**
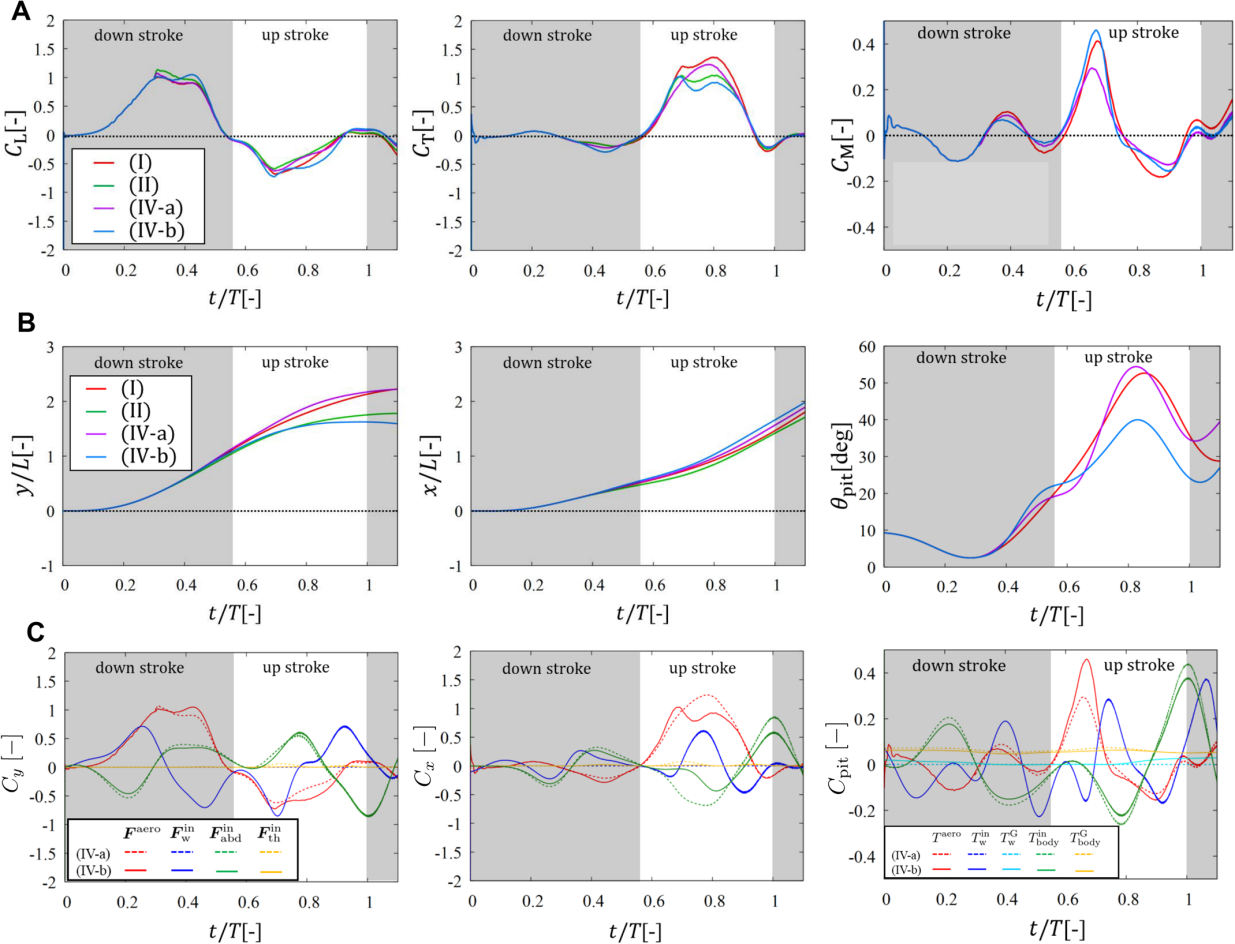
**Results in case iv.** Time variations of (A) the lift coefficient *C*_L_, thrust coefficient *C*_T_, and moment coefficient *C*_M_, and (B) the position of the reference point in the *y*- and *x*-directions and the pitching angle *θ*_pit_ in cases i, ii, iv-a, and iv-b. (C) Comparison of the vertical-force, horizontal-force, and pitching-torque coefficients *C*_*y*_, *C*_*x*_, and *C*_pit_ for the aerodynamic contribution with those for the inertial and gravitational contributions of the wings and body in cases iv-a and iv-b.

Finally, we show the results in case iv to check the effects of the abdominal oscillation and wing mass on the rotational motion of the body. Case iv has two subcases, iv-a and iv-b, which correspond to the cases without and with wing mass, respectively (see also Table 3). Fig. 4 shows the results of the aerodynamic pitching moment coefficient *C*_M_ and pitching angle *θ*_pit_ as well as *C*_L_, *C*_T_, and ***x***=(*x*, *y*) in cases i, ii, iv-a, and iv-b. This figure also includes the comparison between the aerodynamic, inertial, and gravitational forces and torques in cases iv-a and iv-b. From the left and middle panels of Fig. 4A–C and Table 1, we can see that the results in cases iv-a and iv-b have the same tendency as those in cases iii-a and iii-b. That is, the inertial forces of the abdomen and wings are comparable in magnitude with the aerodynamic forces, but the net influence of the inertial forces on the position of the butterfly is not significant due to the offsetting of the body and wing inertia.

Meanwhile, we can see from the right panel of Fig. 4A that the peak values of *C*_M_ in cases i and iv-b are comparable to each other and significantly larger than in cases iv-a. In the right panel of Fig. 4B, however, the peak values of *θ*_pit_ in cases i and iv-a are comparable to each other and significantly larger (by more than 10^°^) than in cases iv-b. The quantitative differences between these cases can be seen in Table 1. This is because the inertial torque 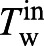 by the wings has large negative peaks around the boundary between the downstroke and upstroke as shown in the right panel of Fig. 4C.

Whereas the inertial forces 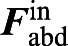 and 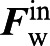 of the body and wings are almost balanced (left and middle panels of Fig. 4C), the inertial torques 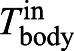 and 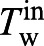 of the body and wings are not balanced (right panel of Fig. 4C). The net influence of the inertial torques is negative and suppresses the increase in the pitching angle *θ*_pit_ during the upstroke (right panel of Fig. 4B). In addition, from the right panel of Fig. 4C, we can see that the gravitational torque 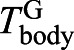 of the body is always positive and almost constant, whereas the gravitational torque 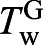 of the wings is small enough to be ignored compared with the other torques. Thus, the net aerodynamic and gravitational torques raise the thorax of the butterfly, and the net inertial torques suppress the rise of the thorax. This scenario is consistent with the result by Sunada et al. (1993).

Finally, we discuss the deviation between cases i and iv-b. In case iv-b, both the position ***x*** and pitching angle *θ*_pit_ are calculated from the equations of motion of the butterfly model. Thus, case iv-b is the most realistic case in our simulations. The calculated curves of *y*, *x*, and *θ*_pit_ in case iv-b have similar shapes to the measured curves in case i. However, there is some non-negligible deviations between cases i and iv-b, especially in the peak value of *θ*_pit_. The deviation in *θ*_pit_ suggests that in case iv-b the aerodynamic and gravitational torques are underestimated or the inertial torques are overestimated due to some modelling errors. A probable candidate of such errors is the assumption that the wing area is constant throughout the take-off. In general, butterflies have partially-overlapping fore- and hindwings and can extend their wings by the lead–lag motion. Thus, the wing area can vary effectively. The variation of the wing area should have a significant influence on the aerodynamic force and torque. In particular, the aerodynamic torque should proportionally increase with increasing distance between the reference point and wing tip, indicating that the aerodynamic torque is quite sensitive to the wing extension. Therefore, the wing extension might be required to improve the accuracy of the rotational motion of the butterfly model. Alternative possibility of the errors in the rotational motion is the assumption that the wings are of constant density (see the Appendix). When we cut a wing into wing-root and wing-tip parts and measured each mass per each area, the wing-root part was heavier than the wing-tip part (by about 40%). Thus, the wing-mass density was biased toward the wing root. This is likely to reduce the moment of inertia of the wings compared with that with uniform mass density. Therefore, we should measure the wing-mass density more accurately and then incorporate it into the butterfly model to improve the accuracy of the rotational motion of the model. These factors will be modeled in future work.

## DISCUSSION

We revisited to flight dynamics in the take-off of a butterfly through experimental measurements and CFD simulations for a cabbage white butterfly (*Pieris rapae*). We measured the motions of characteristic points on the wings and body of the butterfly in take-off and obtained the time series data of the trajectory of the body, pitching angle of the thorax, relative angle of the abdomen to the thorax (abdominal angle), and joint angles of the wings. We constructed a dynamical model composed of a thorax, an abdomen, and four wings (left and right wings with fore and hind parts), i.e. a six-link, rigid-body system. The present butterfly model is more sophisticated than the models which have ever constructed in existing studies. In this model, the body trajectory and thoracic pitching angle can be calculated from the equations of motion, whereas the abdominal angle and wings’ joint angles are prescribed by the measured data. We calculated the flow field and aerodynamic force and torque generated by the butterfly model using the immersed boundary–lattice Boltzmann method. In the present simulations, we considered the motion of the butterfly model in four cases shown in Table 3.

In case i, we prescribe the body trajectory and thoracic pitching angle in addition to other joint angles. The butterfly generates the horizontal and vertical vortex rings behind it during the downstroke and upstroke, respectively. This vortex structure is consistent with the other experimental visualizations (Johansson and Henningsson, 2021). Also, the butterfly generates lift force in the downstroke and thrust force in the upstroke. The vortex structure and aerodynamic forces are resulted from the fact that the butterfly flaps its wings downward in the downstroke and upward in the upstroke by changing the pitching angle, which is consistent with the conclusion of Sunada et al. (1993).

In case ii, we calculate the body trajectory by assuming that all the masses of the model are concentrated at the center of mass. The butterfly can fly upward against the gravity even without leg impulsion, but its vertical position is significantly lower than the experimental data as well as the calculated result with leg impulsion. Thus, the leg impulsion is essential in the upward motion of the taking-off butterfly rather than the aerodynamic lift force by the flapping wings, which is consistent with the conclusion of Bimbard et al. (2013).

In case iii, we calculate the body trajectory from the equations of motion, whereas the thoracic pitching angle is prescribed. The butterfly can fly with almost the same trajectory as in case ii. The inertial forces of the abdomen and wings are comparable in magnitude with the aerodynamic forces, but the net influence of the inertial forces on the position of the butterfly is not significant due to the offsetting of the body and wing inertia. This is consistent with the conclusion of Chang et al. (2020).

In case iv, we calculate both the body trajectory and thoracic pitching angle from the full equations of motion. The butterfly can fly with almost the same trajectory as in case i, but the peak value of the pitching angle is significantly lower than in case i. The inertial torques of the abdomen and wings are comparable in magnitude with the aerodynamic torque, and they do not cancel out unlike the inertial forces. The net aerodynamic and gravitational torques raise the thorax of the butterfly, and the net inertial torques suppress the rise of the thorax. This is consistent with the result of Sunada et al. (1993). The deviation in the thoracic pitching angle between cases i and iv might be attributed to the assumptions that the wing area is constant throughout the take-off and that the wings are of constant density.

## MATERIALS AND METHODS

### Experimental measurements of a butterfly's motion

In the present study, we use cabbage white butterflies (*Pieris rapae*) captured around the Saigawa River in Nagano, Japan. The reasons why this species was selected are that it is easy to find and capture them for a long period in Japan (from March to October) and that it is easy to mark on their white wings. We selected one individual whose weight is comparable to the individuals used by Bimbard et al. (2013). The room air temperature and humidity were 24.9°C and 46.0%, respectively. The physical properties of the butterfly and experiment environment are shown in Table 2. The detailed definitions of these properties are given below.

**Table 2.**
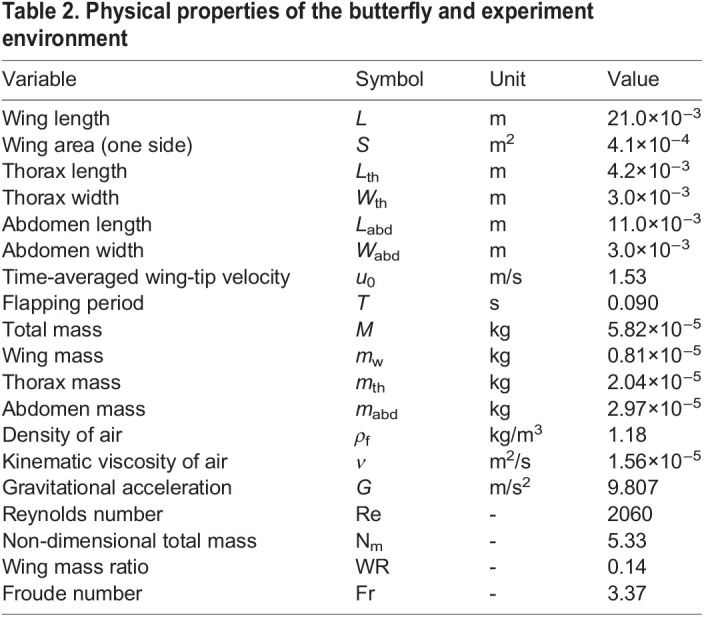
Physical properties of the butterfly and experiment environment

**Fig. 5.**
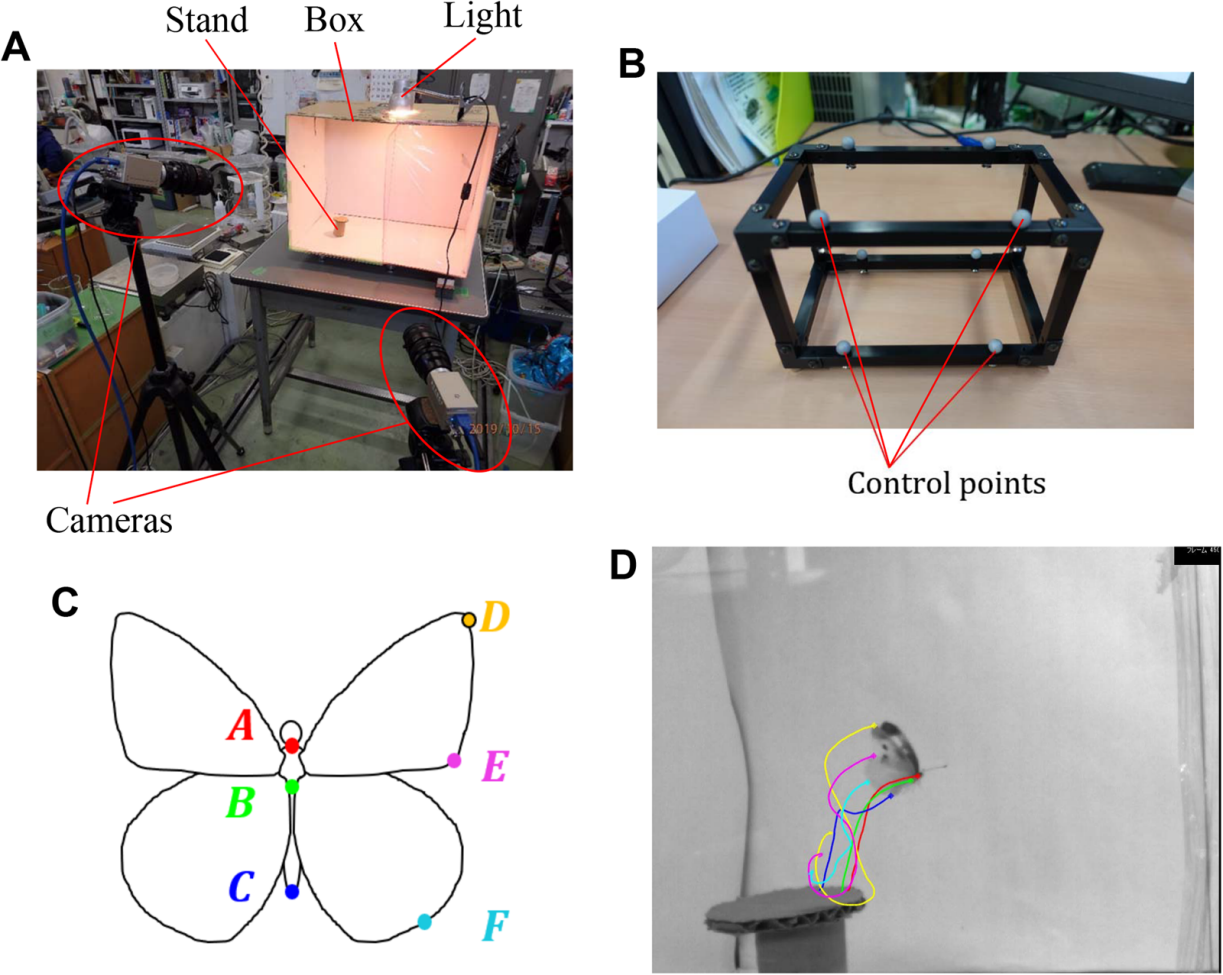
(A) Experimental apparatus; (B) calibrator for motion capturing; (C) monitor points on a butterfly; (D) trajectories of the monitor points.

The experimental apparatus is composed of a box (570×435×375mm^3^, made of corrugated cardboard), a cardboard stand from which the butterfly takes off, a 60W light fixed on the roof of the box, and two high speed video cameras (HAS-U2, DITECT Co. Ltd., Japan) supported by tripods as shown in Fig. 5A. One side of the box is replaced by an acrylic transparent board to take videos through it. The inside walls (except the acrylic board) of the box are covered by white paper to make it clear to observe the butterfly. We use a motion capturing software (DIPP-MotionV/3D, DITECT Co. Ltd., Japan) with a rectangular calibrator. The calibrator has eight control points which are set at the vertex of a 100 mm cube (Fig. 5B). We can calibrate the cameras by taking a video of the eight control points and by inputting the positions of the control points to the motion capturing software. By this calibration procedure, the internal/external parameters of the cameras are determined. In addition, the motion capturing software has functions to trace the monitor points on moving objects from a video, to output two-dimensional positional data of the monitor points, and to convert two sets of the two-dimensional positional data obtained by two synchronized cameras into three-dimensional positional data.

We define six monitor points on the butterfly (Fig. 5C), where the points E and F are marked by a red felt pen. We measure the total mass of the butterfly before recording, and we measure the masses of parts (thorax, abdomen, and wings) after recording. We softly put the butterfly on the stand in the box and wait for its spontaneous take-off. We take movies of the take-off flight by the two synchronized cameras (1000 fps, 640×480 pixel resolution) from a different direction. From the movies, we trace the monitor points by using the motion-capturing software (Fig. 5D; Movie 3). Although the motion-capturing software can trace the monitor points automatically, it occasionally fails when the monitor points on the body are hidden by the wings. In this case, we manually trace these monitor points from the sequential video frames. Finally, we can obtain the three-dimensional positional data of the monitor points by the motion capturing software. Since the obtained data are discretized in time, we convert them into the continuous data by a 17^th^-order polynomial using the least squares approximation.

From the three-dimensional positional data of the six monitor points, we calculate the yawing angle *θ*_yaw_, pitching angle *θ*_pit_, and abdominal angle *θ*_abd_. In addition, we calculate the flapping angle *θ*_flap_ to define the downstroke and upstroke.

Let the position vectors of the six monitor points A–F (Fig. 5C) at time *t* be ***A***(*t*), ***B***(*t*), ***C***(*t*), ***D***(*t*), ***E***(*t*), ***F***(*t*), respectively. These vectors are described as the components for the *x*-, *y*-, and *z*-axes which are fixed to the space, and we define the *y*-axis as the upward vertical direction. Also, let the position vector of the center of the thorax be ***x***_c_(*t*)=[***A***(*t*)+***B***(*t*)]/2. The relative position vectors of the monitor points from the center of the thorax are given by ***X***_c_(*t*)=***X***(*t*)−***x***_c_(*t*), where ***X*** is a representative symbol of ***A***–***F***.

The yawing angle is the angle of the vector ***A***_c_(*t*) projected onto the *zx*-plane from the *x*-axis positive direction. Since the projected vector can be expressed by (*a*_*x*_(*t*), 0, *a*_*z*_(*t*))^T^ (where the superscript T denotes the transpose), the yawing angle *θ*_yaw_(*t*) is given by
(1)

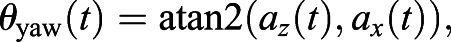
where the function atan2 is the 2-argument arctangent defined in the Appendix. For the next step to derive the pitching angle, we rotate the vectors ***X***_c_(*t*) by *θ*_yaw_(*t*) around the *y*-axis and obtain ***X***_yaw_(*t*)=*S*_2_(*θ*_yaw_(*t*))***X***_c_(*t*), where *S*_*i*_ (*i*=1, 2, 3) is the rotational matrix around the *i*th axis shown in the Appendix. By this rotation, the points A, B, and C on the body are put on the *xy*-plane.

The pitching angle is the angle of the vector ***A***_yaw_(*t*) from the *x*-axis positive direction. Since ***A***_yaw_(*t*) can be expressed by (*b*_*x*_(*t*), *b*_*y*_(*t*), 0)^T^, the pitching angle *θ*_pit_ is given by
(2)

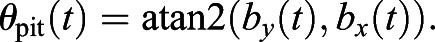


For the next step to derive the abdominal angle, we rotate the vectors ***X***_yaw_(*t*) by *θ*_pit_(*t*) around the *z*-axis and obtain ***X***_pit_(*t*)=*S*_3_(−*θ*_pit_(*t*))***X***_yaw_(*t*). By this rotation, the points A and B on the thorax are put on the *x*-axis.

The abdominal angle is the angle of the vector ***C***_pit_(*t*)−***B***_pit_(*t*) from the *x*-axis negative direction. Since ***C***_pit_(*t*)−***B***_pit_(*t*) can be expressed by (*c*_*x*_(*t*), *c*_*y*_(*t*), 0)^T^, the abdominal angle *θ*_abd_(*t*) is given by
(3)

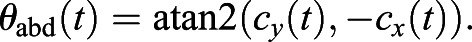


It is noted that the abdomen rotates in the dorsal direction for *θ*_abd_>0 (see Fig. 7C).

The flapping angle defined here is the angle between the *zx*-plane and the right forewing. In other words, the flapping angle is given as the angle between the *y*-axis negative direction and the normal vector (ventral side) of the right forewing projected onto the *yz*-plane. Since the right forewing is expressed by the plane spanned by ***D***_pit_(*t*) and ***E***_pit_(*t*), its normal vector is given by ***n***(*t*)=***D***_pit_(*t*)×***E***_pit_(*t*)=(*n*_*x*_(*t*), *n*_*y*_(*t*), *n*_*z*_(*t*))^T^. Thus, the flapping angle *θ*_flap_ is given by
(4)

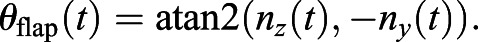
In this definition, the wings are at the top and bottom dead points when *θ*_flap_ reaches the maximum and minimum, respectively.

**Fig. 6.**
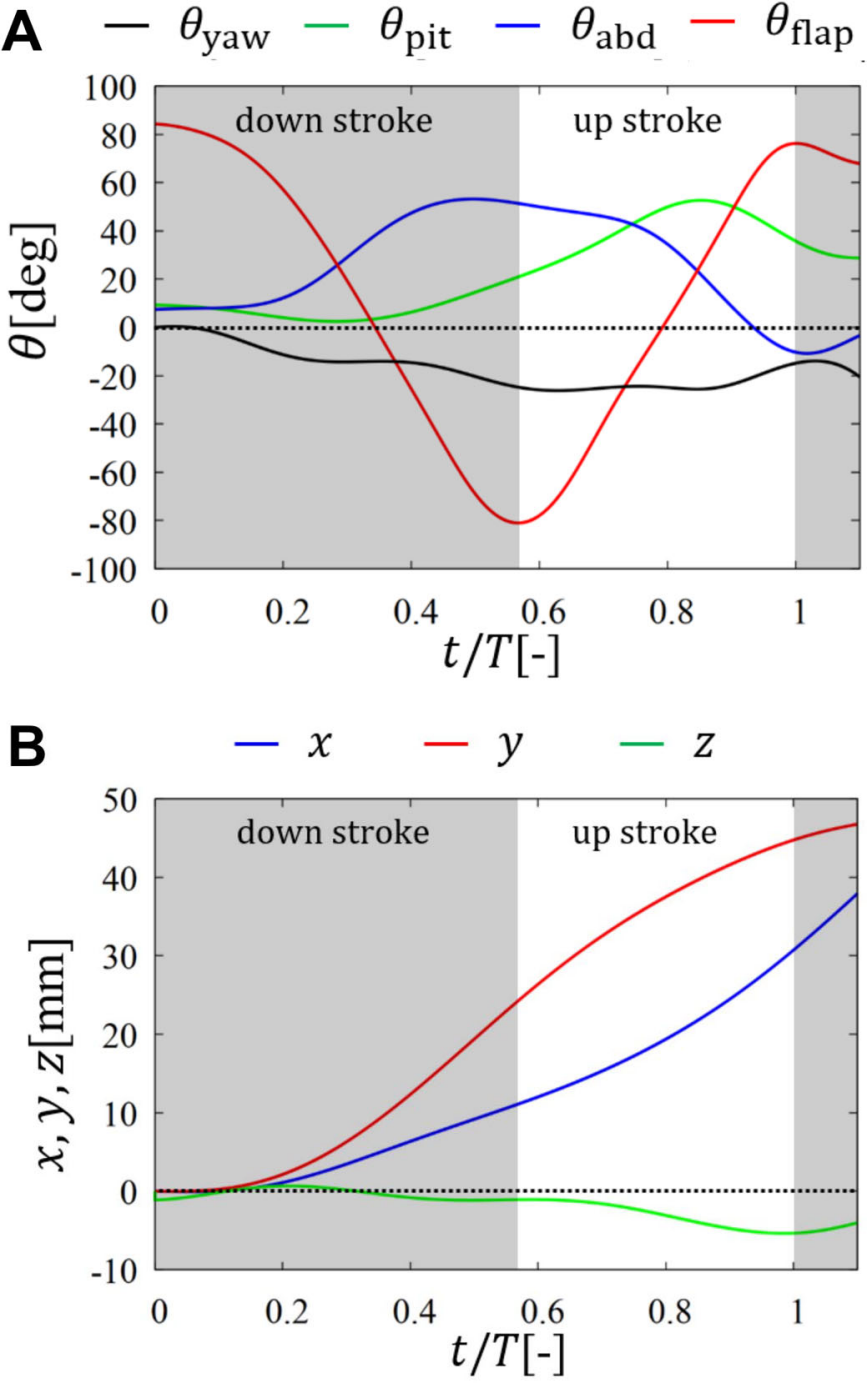
**Motion of the body of a butterfly during one period after the take-off.** (A) Time variations of the yawing angle *θ*_yaw_, the pitching angle *θ*_pit_, the abdominal angle *θ*_abd_, and the flapping angle *θ*_flap_; (B) trajectories of the monitor point B where *x* is the forward direction and *y* is the vertical direction. In A, the flapping angle *θ*_flap_ is used to define the downstroke and upstroke.

Fig. 6 shows the motion of the body of the butterfly during one period after the take-off. We define the downstroke and upstroke as the periods during which *θ*_flap_ varies from the maximum to the minimum and from the minimum to the maximum, respectively. The time when *θ*_flap_ reaches the first maximum is set to *t*=0 s. The flapping period (i.e. the period during which *θ*_flap_ varies from the first maximum to the second maximum) is *T*=0.090 s. The horizontal axis of Fig. 6 is normalized by the flapping period *T*. It is noted that we initially rotate and translate the space-fixed coordinate system so that *θ*_yaw_(0)=0^°^ and ***B***(0)=***0*** mm.

We can see from Fig. 6 that the yawing angle *θ*_yaw_ and *z*-displacement slightly deviate from zero at most about − 20^°^ and − 5 mm, respectively. Thus, the butterfly does not turn after taking off and flies almost in the longitudinal plane (*xy*-plane). As for the relationship among the pitching angle *θ*_pit_, abdominal angle *θ*_abd_, and flapping angle *θ*_flap_, we can see from Fig. 6A that *θ*_pit_ has the minimum and maximum around the middle of the downstroke and the middle of the upstroke, respectively, whereas *θ*_abd_ has the minimum around the start of the downstroke and the maximum around the end of the downstroke. Thus, *θ*_pit_ and *θ*_abd_ oscillate almost the same frequency as that in *θ*_flap_, and the phase differences of *θ*_pit_ and *θ*_abd_ from *θ*_flap_ are about *π*/2 and *π*, respectively. A similar relationship can be observed in the forward flight of a paper-kite butterfly (*Idea leuconoe*) reported by Chang et al. (2020). Due to the phase difference between *θ*_pit_ and *θ*_flap_, the butterfly flaps its wings downward in the downstroke and backward in the upstroke.

It should be noted that the measured motion of the butterfly in Fig. 6 is a typical one among other measured motions (sample size is *n*=8) as shown in Fig. S1.

### Numerical modeling

**Fig. 7.**
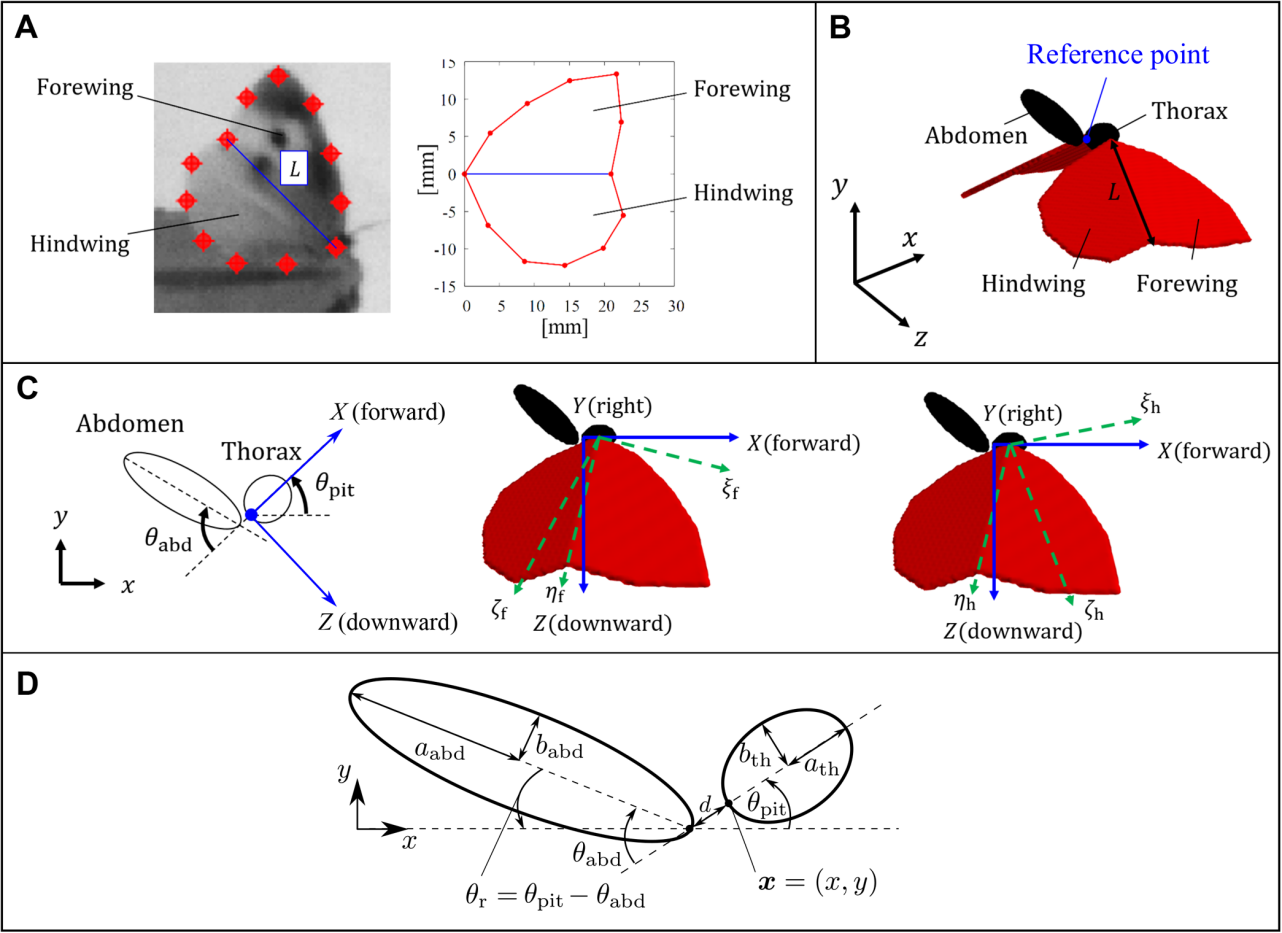
**Numerical modeling of a butterfly.** (A) Shape of the right fore- and hindwings; (B) butterfly model consisting of two fore- and hindwings, an ellipsoidal thorax, and an ellipsoidal abdomen; (C) relationship between the axes fixed to the space *x*–*y*–*z* (Σ_s_), fixed to the thorax *X*–*Y*–*Z* (Σ_th_), fixed to the right forewing *ξ*_f_–*η*_f_–*ζ*_f_ (Σ_rfw_), and fixed to the right hindwing *ξ*_h_–*η*_h_–*ζ*_h_ (Σ_rhw_); (D) thorax–abdomen system in the butterfly model (see the Appendix).

Here, we construct a numerical model of the butterfly. The wing shape is captured from the video frame at *t*=0 s (the time when the wings are at the first top dead point). We measured the positions of 12 points on the outline of the right wing (left panel of Fig. 7A), and numerically reconstructed the outline by the line segments connecting these points (right panel of Fig. 7A). It should be noted that the model wing can be flexed on the blue line in Fig. 7A. Thus, the fore- and hindwings are approximately distinguished in the present model. We define the wing length *L* as the length of the blue line. We assume that the left wing has the same shape as the right wing. The model wings are rigid and infinitely thin.

The present butterfly model is composed of the thorax, abdomen, right fore- and hindwings, and left fore- and hindwings as shown in Fig. 7(B). The model thorax and abdomen are expressed by the ellipsoids with major axis length *L*_th_ and *L*_abd_ and with minor axis length *W*_th_ and *W*_abd_, respectively. These lengths were measured from the video frame at the time when the body of the butterfly can be clearly observed. To prevent the parts of the model from colliding with other parts, we set a short distance 0.05*L* between the thorax surface and wing root as well as between the end-point nodes of the thorax and abdomen. By measuring the masses of the parts of the butterfly, we found that the center of mass of the body is located at the connection point (monitor point B) between the thorax and abdomen when the body is straight. We regard this point as the reference point of the body of the model (Fig. 7B,C).

To describe the motions of the body and the wings, we introduce the following four coordinate systems. First, let the coordinate system fixed to the space be Σ_s_. The axes of Σ_s_ are denoted by *x*, *y*, and *z*, where the *x*- and *y*-axes are the forward and vertically upward directions, respectively (Fig. 7B). Thus, the *xy*-plane is the longitudinal plane. Second, let the coordinate system fixed to the thorax be Σ_th_, and its origin be located at the reference point. The axes of Σ_th_ are denoted by *X*, *Y*, and *Z*, where the *X*- and *Z*-axes are the forward and downward directions relative to the thorax, respectively (left panel in Fig. 7C). Third, let the coordinate system fixed to the right forewing be Σ_rfw_, and its origin be located at the wing root and fixed to (*X*, *Y*, *Z*)=(0.5*L*_th_, 0.5*W*_th_+0.05*L*, 0) in Σ_th_. The axes of Σ_rfw_ are denoted by *ξ*_f_, *η*_f_, and *ζ*_f_, where the *η*_f_-axis is parallel to the line which distinguishes the fore- and hindwings, and the *ζ*_f_-axis is perpendicular to the forewing in the dorsal direction (middle panel in Fig. 7C). Forth, let the coordinate system fixed to the right hindwing be Σ_rhw_, and its origin be the same as that of Σ_rfw_. The axes of Σ_rhw_ are denoted by *ξ*_h_, *η*_h_, and *ζ*_h_, where the *η*_h_-axis is the same as the *η*_f_-axis, and the *ζ*_h_-axis is perpendicular to the hindwing in the dorsal direction (right panel in Fig. 7C). We assume that the motion of the left wing is symmetrical to the right wing about the *ZX*-plane. Thus, the definition of the coordinate system fixed to the left wing is not required.

We assume that the body motion is restricted in the longitudinal plane, i.e. the model does not have either the yawing or rolling angle. The coordinate system Σ_th_ fixed to the thorax is described by the pitching rotation relative to the coordinate system Σ_s_ fixed to the space. Let ***e***_*x*_, ***e***_*y*_, and ***e***_*z*_ be three unit vectors along the *x*-, *y*-, and *z*-axes, respectively, and ***e***_*X*_, ***e***_*Y*_, and ***e***_*Z*_ be three unit vectors along the *X*-, *Y*-, and *Z*-axes, respectively. The vector array [***e***_*X*_, ***e***_*Y*_, ***e***_*Z*_] is given by the orthogonal transformations of [***e***_*x*_, ***e***_*y*_, ***e***_*z*_] as follows:
(5)

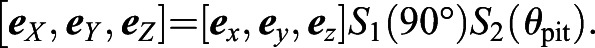


Also, the abdomen rotates relative to Σ_th_. An end-point node of the abdomen is fixed to (*X*, *Y*, *Z*)=(−0.05*L*, 0, 0), and the abdomen rotates around the *Y*-axis by the abdominal angle *θ*_abd_ in the clockwise direction (left panel in Fig. 7C).

The coordinate system Σ_rfw_ fixed to the right forewing is described by the rotations relative to Σ_th_. Let 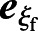, 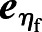, and 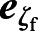 be three unit vectors along the *ξ*_f_-, *η*_f_-, and *ζ*_f_-axes, respectively. The vector array 
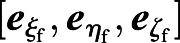
 is given by the successive orthogonal transformations of [***e***_*X*_, ***e***_*Y*_, ***e***_*Z*_] using the 3–1–2 Euler angle as follows:
(6)

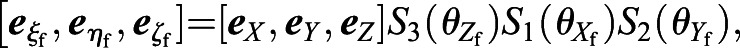
where the three angles 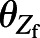, 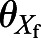, and 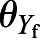 are determined by the positional vectors ***D***_pit_(*t*) and ***E***_pit_(*t*) (see also the previous subsection). At first, we consider the vector ***E***_pit_(*t*) projected onto the *XY*-plane, which is expressed by (*d*_*x*_(*t*), *d*_*y*_(*t*), 0)^T^. The angle 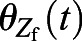 is given by
(7)

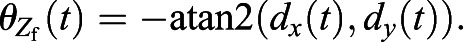


Secondly, to derive the angle 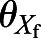, we rotate ***D***_pit_(*t*) and ***E***_pit_(*t*) around the *Z*-axis by the angle 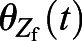, and we obtain 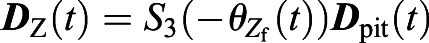 and 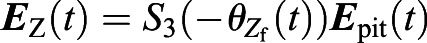. The vector ***E***_*Z*_(*t*) can be expressed by (0, *e*_*y*_(*t*), *e*_*z*_(*t*))^T^, and the angle 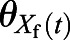 is given by
(8)

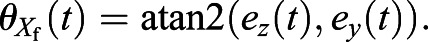


Finally, to derive the angle 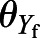, we rotate ***D***_*Z*_(*t*) around the *X*-axis by the angle 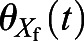, and we obtain 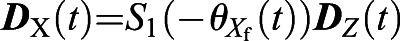. We consider the vector ***D***_*X*_(*t*) projected onto the *ZX*-plane, which is expressed by (*f*_*x*_(*t*), 0, *f*_*z*_(*t*))^T^. The angle 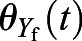 is given by
(9)

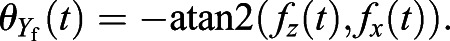


It should be noted that the angles 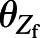, 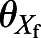, and 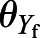 do not correspond to the flapping, feathering, or lead-lag angle. We use these angles to properly reproduce the wing motion of the butterfly.

Also, the coordinate system Σ_rhw_ fixed to the right hindwing is described by the rotations relative to Σ_th_ using the 3–1–2 Euler angle. The angles 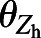, 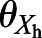, and 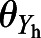 for the hindwing are determined by the positional vectors ***E***_pit_(*t*) and ***F***_pit_(*t*) in the same way as the forewing. It should be noted that the first and second angles are the same as those for the forewing, i.e. 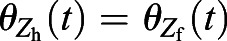 and 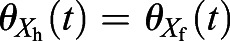. The angle 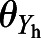 is different from 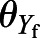 and derived from 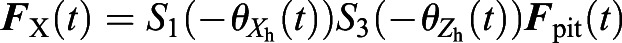 instead of ***D***_*X*_(*t*).

In summary, the motion of the present model is described by the following variables: the position of the reference point ***x***=(*x*, *y*, 0)^T^ observed in Σ_s_; the pitching angle *θ*_pit_; the abdominal angle *θ*_abd_; the joint angles for the forewing 
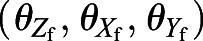
 and for the hindwing 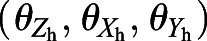. The variables ***x*** and *θ*_pit_ can be obtained from the equations of motion, and the other variables are prescribed. When we require the time derivatives of the prescribed variables, we calculate them by using the first-order backward difference approximation.

### Equations of motion and numerical method

#### Motion of the fluid

The fluid motion around the butterfly model is governed by the continuity equation and Navier–Stokes equations for an incompressible fluid:
(10)

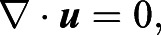

(11)

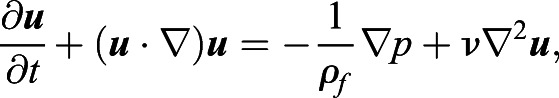
where ***u*** is the fluid velocity, *p* is the pressure, *ρ*_f_ is the fluid density, and *ν* is the kinematic viscosity of the fluid. The no-slip condition must be satisfied on the surface of the model, i.e. the fluid velocity must be equal to the velocity of the wings and body.

We calculate the above equations by using the lattice Boltzmann method and enforce the no-slip condition by using the immersed boundary method. The wings and body of the model are represented by an arrangement of boundary Lagrangian points. The position and velocity of the boundary Lagrangian points on the wings and body are updated by orthogonal transformation of the coordinate systems fixed to the wings and body relative to that fixed to the space. For details of the numerical method, see Suzuki and Inamuro (2011). The validation of the numerical method has been extensively checked in Suzuki et al. (2015).

In this study, we define the mean wing-tip speed as
(12)

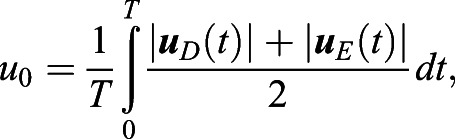
where ***u***_D_(*t*) and ***u***_E_(*t*) are the velocities of the monitor points D and E relative to the body which are given by the first-order difference approximation of ***D***_pit_(*t*) and ***E***_pit_(*t*), respectively. The governing parameter of the above equations is the Reynolds number Re given by
(13)

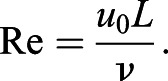


#### Motion of the butterfly model

As mentioned in the introduction, we consider the motion of the butterfly model in the following four cases: (i) to prescribe both the translational and rotational motions by the experimental data; (ii) to calculate the translational motion of the model by assuming that all the masses of the model are concentrated at the reference point; (iii) to calculate the translational motion of the thorax from the equations of motion, whereas the rotational motion is prescribed; (iv) to calculate both the translational and rotational motions of the thorax from the full equations of motion.

In case i, there is no need to calculate the equations of motion of the model, and both the position of the reference point ***x***=(*x*, *y*, 0)^T^ and the pitching angle *θ*_pit_ are prescribed.

In case ii, the position of the reference point is calculated from the Newton equation as follows:
(14)

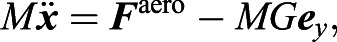
where the dot notation denotes the time derivative, ***F***^aero^ is the aerodynamic force acting on the model, *M* is the total mass of the model, and *G* is the gravitational acceleration. On the other hand, the pitching angle is prescribed.

In case iii, we formulate the Lagrange equations for the thorax–abdomen–wings system. It should be noted that since the Lagrangian for the wings is difficult to be formulated, we formulate the Lagrangian for the thorax and abdomen and compute the effect of the wing inertia as the external force. Letting the Lagrangian for the thorax and abdomen be 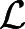, the Lagrange equations for the model are given by
(15)

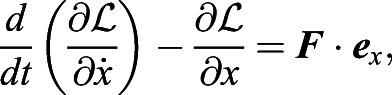

(16)

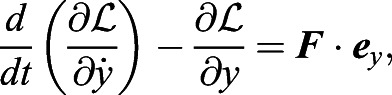
where ***F*** is the force acting on the model including the aerodynamic force, gravitational force, and inertial force of the wings. Thus, ***F*** is given by
(17)

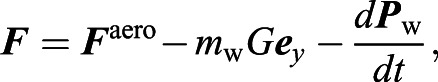
where *m*_w_ is the mass of the wings, and ***P***_w_ is the linear momentum of the wings, which is computed as shown in the Appendix. On the other hand, the pitching angle is prescribed.

In case iv, we need the following equation for the pitching angle *θ*_pit_ in addition to Eqns (15) and (16):
(18)

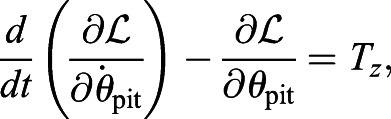
where *T*_*z*_ is the torque (in the *z*-direction) acting on the model around the reference point including the aerodynamic torque, gravitational torque, and inertial torque of the wings. Thus, *T*_*z*_ is given by
(19)

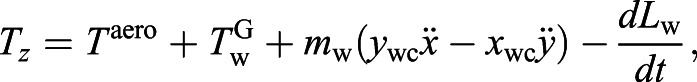
where *T*^aero^ is the aerodynamic torque acting on the model, 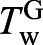 is the gravitational torque of the wings, (*x*_wc_, *y*_wc_, *z*_wc_)^T^ is the position of the center of mass of the wings relative to the reference point observed in Σ_s_, and *L*_w_ is the angular momentum of the wings around the reference point, which is computed as shown in the Appendix.

As for the numerical method, we use the second-order Adams–Bashforth method to solve Eqn (14) in case ii. In cases iii and iv, we reform Eqns (15), (16), and (18) into the matrix form (see the Appendix and Fig. 7D), and we solve it by using the second-order Adams–Bashforth method. The aerodynamic force ***F***^aero^ and torque *T*^aero^ are calculated by the summation of the volume force which is applied to enforce the no-slip condition on the model surface in the immersed boundary method. In addition, since the thorax and abdomen have volume, the internal mass effect (Suzuki and Inamuro, 2011) must be considered for the aerodynamic force and torque acting on the body. The internal mass effect for the body is calculated by using the Lagrangian points approximation. On the other hand, the internal mass effect for the wings is neglected, since the wings have no volume. It should be noted that in our preliminary calculations, the aerodynamic force and torque acting on the body are much smaller than those on the wings.

The governing parameters of this system are the non-dimensional mass (N_m_), the wing-mass ratio (WR), and the Froude number (Fr) defined as:
(20)

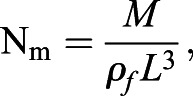

(21)

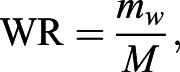

(22)

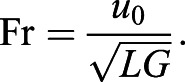


The cases in the present simulations are summarized in Table 3. The subcases ‘a’ and ‘b’ denote the cases without and with wing mass, respectively, to check the effects of the wing mass on the translational and rotational motions of the body. It should be noted that in both subcases the total mass of the model is unchanged.

**Table 3.**
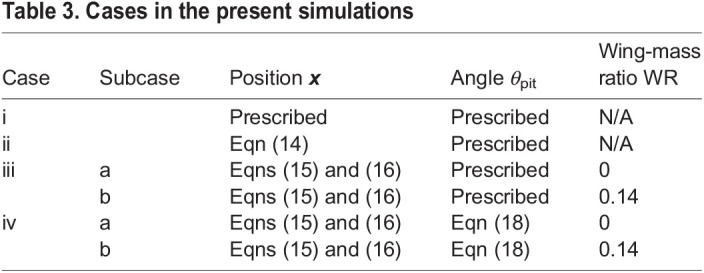
Cases in the present simulations

### Computational conditions and parameters

The computational domain is a cube with a side length equal to *W*=12*L*. The boundary condition on the two sides perpendicular to the *x*-axis is the periodic boundary condition, and on the other sides the no-slip condition is used. The inner fine grid (Inamuro, 2012) used only around the model is a cube with a side length equal to 3*L*. The reference point is initially placed at the center of the domain, and the fluid in the domain is initially stationary and at a uniform pressure. To reduce the computational cost, we calculate one-half of the computational domain with the mirror boundary condition on the longitudinal plane which passes through the center of the domain and is perpendicular to the *z*-axis.

Since computations of three-dimensional moving boundary flows at the measured value of the Reynolds number (Re=2060) are quite expensive, and aerodynamic force and moment coefficients generated in butterfly's flight are relatively insensitive to the Reynolds number (Yokoyama et al., 2013), we set Re=1000 by using about 2 times larger kinematic viscosity. It should be noted that the other non-dimensional governing parameters are the same as those obtained by the experiment shown in Table 2. The spatial and temporal resolutions are set to *L*=120Δ*x* and *T*=15840Δ*t*, where Δ*x* and Δ*t* are the lattice spacing and time step, respectively.

In the take-off of a butterfly from the ground, the leg impulsion due to the active leg extension is important for the initiation of the upward motion of the butterfly's body (Bimbard et al., 2013). In the present study, we include this impulsion by prescribing the motion of the model until *t*/*T*=0.3 at which in the experiment the butterfly's legs were observed to leave the stand. Thus, in the early stage of the simulation 0≤*t*/*T*≤0.3, the results in cases ii–iv coincide with the result in case i. After that, the motion of the model is calculated as explained in the previous subsection.

## APPENDIX

### Functions used in calculating joint angles

In calculating the joint angles from the position vectors of the six monitor points, we use the following functions. To avoid the singularity and discontinuity of the arctangent, we use the two-argument arctangent defined as

(23)

In addition, we use the rotational matrices *S*_1_, *S*_2_, and *S*_3_ given as

(24)

(25)

(26)

### Supplements to equations of motion of the butterfly model

Here, we formulate the equations of motion of the butterfly model in detail. Undefined variables in this section can be found in Table 2.

### Lagrangian for thorax and abdomen

The body of the present butterfly model is composed of an ellipsoidal thorax and an ellipsoidal abdomen as shown in Fig. 7(D). The semi-major and semi-minor axes of the thorax are respectively denoted by *a*_th_=*L*_th_/2 and *b*_th_=*W*_th_/2, and those of the abdomen are denoted by *a*_abd_=*L*_abd_/2 and *b*_abd_=*W*_abd_/2. The distance between the end-point nodes of the thorax and abdomen is set to *d*=0.05*L*. The reference point ***x***=(*x*,*y*) is located at the thoracic end-point node closer to the abdomen. The pitching angle *θ*_pit_ is defined as the angle of the thoracic major axis with respect to the horizontal line, and the abdominal angle *θ*_abd_ is defined as the relative angle between the thoracic and abdominal major axes. Note that *θ*_pit_ is positive when the thorax rotates in the counter-clockwise direction, whereas *θ*_abd_ is positive when the abdomen rotates in the clockwise direction.

The Lagrangian for the thorax and abdomen of the butterfly model, which is used in the Lagrange Eqns (15), (16), and (18), is given by

(27)

where *θ*_r_=*θ*_pit_−*θ*_abd_ is the angle of the abdominal major axis with respect to the horizontal line, and the dot notation denotes the time derivative.

### Inertial force of wings

In cases iii-b and iv-b, we have to calculate the time derivatives of the linear momentum ***P***_w_ in Eqn (17). It should be noted that in the present study we assume that the wings are of constant density for simplicity.

Denoting the density of the wings by *ρ*_w_=*m*_w_/*S* and the positional vector of an arbitrary point on the wings by ***x***_w_, we can rewrite the time derivative of the linear momentum of the wings in integral formulation as

(28)

where  means the surface integral over the wings. Viewed from the reference point ***x***, the above equation is rearranged as follows:

(29)

where ***x***_wo_=***x***_w_−***x***=(*x*_wo_, *y*_wo_, *z*_wo_)^T^ is the positional vector of an arbitrary point on the wings relative to the reference point observed in the space-fixed coordinate system Σ_s_. The inertial force  in the right-hand side of Eqn (29) is computed using a difference approximation and numerical integration.

### Gravitational and inertial torques of wings

In cases iii-b and iv-b, we have to calculate the gravitational torque , position ***x***_wc_=(*x*_wc_, *y*_wc_, *z*_wc_)^T^ of the center of mass of the wings relative to the reference point observed in Σ_s_, and time derivatives of the angular momentum ***L***_w_=(0, 0, *L*_w_)^T^ of the wings in Eqn (19).

We can write the gravitational torque of the wings in integral formulation as

(30)

We compute the right-hand side of Eqn (30) using a numerical integration.

We can write the position of the center of mass of the wings in integral formulation as

(31)

In the same way as Eqn (30), we compute the right-hand side of Eqn (31) using a numerical integration.

Also, we can write the time derivative of the angular momentum of the wings around the reference point in integral formulation as

(32)

Observed in the thorax-fixed coordinate system Σ_th_, the vector ***x***_wo_ is transformed into  as follows:

(33)

By substituting Eqn (33) into Eqn (32) and considering the component of the pitching rotation, we can obtain

(34)

In the right-hand side of Eqn (34), we compute the inertia moment *I*_w_ and the inertial torque  of the wings using a difference approximation and numerical integration.

### Matrix forms of equations in case iii

In case iii, the position of the reference point ***x***=(*x*, *y*, 0)^T^ is governed by Eqns (15) and (16), whereas the pitching angle *θ*_pit_ is prescribed. We can rearrange Eqns (15) and (16) in matrix from as

(35)

where the mass matrix  in case iii is given by

(36)

the inertial force  of the wings is computed by numerical integration, the inertial force  of the thorax is given by

(37)

and the inertial force  of the abdomen is given by

(38)

In the simulation of case iii, we obtain *x* and *y* by computing Eqn (35) with the second-order Adams–Bashforth method.

### Matrix forms of equations in case iv

In case iv, the position of the reference point ***x***=(*x*, *y*, 0)^T^ is governed by Eqns (15) and (16), and the pitching angle *θ*_pit_ is governed by Eqn (18). We can rearrange Eqns (15), (16), and (18) in matrix from as

(39)

where the mass matrix  in case iv is given by

(40)

It should be noted that in the above equations the values of *x*_wc_, *y*_wc_, and *I*_w_ are computed by a numerical integration. As the terms with  should be involved in the left-hand side of Eqn (39), the inertial forces  and  are changed from those in case iii as follows:

(41)

(42)

As for the torques, the inertial torque  of the wings is computed by a difference approximation and numerical integration, the inertial torque  of the body is given by

(43)

the gravitational torque  of the wings is computed by a numerical integration, and the gravitational torque  of the body is given by

(44)

In the simulation of case iv, we obtain *x*, *y*, and *θ*_pit_ by computing Eqn (39) with the second-order Adams–Bashforth method.
